# Can thiol compounds be used as biomarkers of aquatic ecosystem contamination by cadmium?

**DOI:** 10.2478/v10102-009-0013-3

**Published:** 2009-09-28

**Authors:** Jana Kovářová, Zdeňka Svobodová

**Affiliations:** University of Veterinary and Pharmaceutical Sciences Brno, Faculty of Veterinary Hygiene and Ecology, Department of Veterinary Public Health and Toxicology, 612 42 Brno, Czech Republic

**Keywords:** metallothionein, glutathione, metal pollution, Cd, fish

## Abstract

Due to anthropogenic activities, heavy metals still represent a threat for various trophic levels. If aquatic animals are exposed to heavy metals we can obviously observe considerable toxicity. It is well known that an organism affected by cadmium (Cd) synthesize low molecular mass thiol compounds rich in cysteine (Cys), such as metallothioneins (MT) and glutathione (GSH/GSSG). The aim of this study was to summarize the effect of Cd on level of thiol compounds in aquatic organisms, and evaluate that the concentrations of thiol compounds are effective indicators of Cd water pollution and explain their potential use in biomonitoring applications.

## Introduction

Heavy metals, such as cadmium (Cd), lead (Pb) and mercury (Hg), still represent a health risk to living organisms and their wide distribution is caused in connection with human activity. Cadmium (Cd) is ubiquitous toxicant which has been recognized as one of the most deleterious heavy metals and has been ranked tenth on the European Union list of priority substances (Water Framework Directory). It is a common pollutant in surface waters (Bouraoui *et al*., 2008), and can cause adverse effects on fish and other organisms inhabiting these ecosystems (Bervoets and Blust, 2003). Since chemical monitoring of water or water sediments is technically and financially demanding, there is a serious requirement for measuring specific biomarkers. Biomarkers are considered “early warning” tools in environmental assessment (McCarthy and Shugard, 1990) and provide qualitative measure of exposure to toxic chemicals or environmental stresses (Sarkar *et al*., 2006). Currently, aquatic ecosystems are monitored in long-term studies by measuring concentrations of biochemical markers such as cytochrom P450 (Havelkova *et al*., 2008), ethoxyresorufin-*O*-deethylase (Randak *et al*., 2009), vitellogenin or 1-hydroxypyrene (Blahova *et al*., 2008) in fish to assess environmental pollution by organic pollutants mainly polycyclic aromatic hydrocarbons (PAHY) and polychlorinated biphenyls (PCB). Several studies have concluded that exposure to metal such as Cd cause increase of thiol compounds in living body (Thomas *et al*., 1982; Klaverkamp and Duncan, 1987; Kägi and Schäffer, 1988; Olsson *et al*., 1998; Schlenk and Rice, 1998; Santovito *et al*., 2000; Kovarova *et al*., 2009). Thiols such as metallothioneins (MT) and glutathione (GSH/GSSG) are compounds, which contain sulphydryl (thiol) groups for binding a variety of metals, including Cd (Mason and Jenkins, 1995; Maracine and Segner, 1998). This phenomenon presents the detoxifying system of metals inhibiting their negative incidence. In many species (annelids, molluscs, crustaceans, fish...) induction of MT or metallothionein-like proteins (MTLP) (Santovito *et al*. 2000; Alvarado *et al*. 2005; Amiard *et al*., 2006), as well as GSH/GSSG (Lange *et al*., 2002; Belcastro *et al*., 2009) by metals (Ag, Cd, Cu, Hg) has been demonstrated.

The aim of the present study is to evaluate that the concentrations of thiol compounds are effective indicators of Cd water pollution and explain their potential use in biomonitoring applications.

## Thiol compounds

Among thiol compounds, there are introduced substances containing SH- (thiol) group, which is a part of amino acid cysteine (Cys). This chemical moiety of protein/peptide molecule is competent for binding metals, and the intracellular fate of both essential and nonessential metal ions strongly depends on the level of thiol containing molecules (Eaton *et al*., 1980). The most widely known thiols are GSH/GSSG and MT, which primary function is to sequester excessive amounts of metals and thus protect cells from metal toxicity (Schlenk and Rice, 1998).

**Metallothioneins** are heat-stable, low molecular weight proteins with a high content of cysteinyl residues (up to 30% of total amino acids), and the absence of aromatic and hydrophobic amino acids (Hamer, 1986). These proteins have a conservative structure and are widely distributed, occurring in procaryotes, protists, fungi, plants and animals, both invertebrates and vertebrates (Roesjadi, 1992). There are only a little structural differences, while MTLP name is in same cases used (Won *et al*., 2008). MTs or MTLPs are classified into three classes on the basis of the locations of cysteine residues and mode of synthesis (Fowler *et al*., 1987). In each class of these proteins, there are included many isoforms coexisting within organisms and performing different functions in terms of the protection cells and organism (Amiard *et al*., 2006). They provide a reservoir of nontoxic Cu and Zn for a number of potentially rate-limiting Cu and Zn metalloenzymes, which may modulate many important cellular processes (e.g., replication, respiration, transcription, protein synthesis and degradation, and energy metabolism); and also they limit the nonspecific binding of nonessential metals within the cell and thereby reduce their potential for inducing metal toxicity (Suzuki *et al*., 1993). Basally-synthesized pool of MT to carry out the physiologically functions (Roesjadi, 1992), can be topped by the induced MT synthesis. Induction of *de novo* synthesis of MT has been studied by means of MT mRNA (Lange *et al*., 2002; Bae *et al*., 2005; Woo *et al*., 2006) and is induced by a number of metals, such as mercury (Hg), copper (Cu), silver (Ag), bismuth (Bi), Cd, Pb, zinc (Zn) and cobalt (Co) (Vasak, 1991), whereas nickel (Ni) and chrome (Cr) negatively affect the level of MT mRNA (Woo *et al*., 2006). MT synthesis is also induced by cytokines and stress hormones as well as by a wide range of chemicals, many of which act indirectly via a stress or inflammatory response (Coyle *et al*., 2002). It is also stimulated upon starvation, high or low temperature, exertion, increased level of steroid hormones (Mason and Jenkins, 1995), and the presence of antibiotics, vitamins or herbicides (Baer and Thomas, 1990). Moreover, MT synthesis is also stimulated upon tissue damage resulting from many situations for instance bacterial infections (Smirnov *et al*., 2004).

The tripeptide **glutathione** (γ-L-glutamyl-L-cysteinyl-glycine) is the most abundant low-molecular-weight thiol-containing molecule in the cell, at physiological level performs serious of functions (Belcastro *et al*., 2009). It participates in a number of fundamental cellular processes, including the synthesis of proteins, transport of amino acids, enzyme activity and cell defence against a variety of internal and external stressors (Meister and Anderson, 1983) – it plays an important role of intracellular reducer, maintaining reduced sulfhydryl groups (SH) of the enzymes (Jezierska and Witeska, 2001), and defences against the toxic action of xenobiotics, metal cations as well as oxyradicals (Meister and Anderson, 1983), hence it acts as a free-radicals scavenger in several important non-enzymatic antioxidant processes and thus has shown anticancer activity (Singhal *et al*., 1987). One of the most important reactions of xenobiotic metabolism is conjugation with GSH to produce more water soluble end product that may be easily excreted from the organism. In studies using purified GSH, it has been shown repeatedly, that the divalent cations of Hg, Cd, Zn and Cu avidly complex with GSH (Thomas *et al*., 1982). From studies with mammals, it has been suggested that GSH acts as a first line of defence against metal toxicity (Singhal *et al*., 1987) by binding metals before the induced synthesis of MT establish effective MT levels. It occurs as reduced (GSH) and oxidized (GSSG) forms in organisms, due to its function. After the reduction of enzymatic SH groups, scavenging radicals, or binding metal ions, follows oxidation of reduced GSH to the oxidized disulphide GSSG. Therefore, the GSH/GSSG ratio is often considered as an indicator of the intracellular redox state, with enhanced values of GSSG/GSH pointing to oxidative stress (Lange *et al*., 2002). Since GSH redox cycle is an important antioxidant defence mechanism, one example for an indirect protective effect against metal toxicity consists in reduction of metal-induced oxidative stress (Maracine and Segner, 1998). In connection with metals oxidative stress results also from metal-catalysed redox reactions (Sugiyama, 1994), and it is succumbed under GSH reduction, too. Besides, this sulphydryl-rich tripetide can interfere with toxic metals in other various ways: it can alter rates of metal uptake and elimination (Foulkes, 1993), it can act as an intracellular metal chelator, thereby preventing nucleophilic interaction of metal ions with essential cellular structures (Meister, 1985), it can assist in the maintenance of cellular Ca^2+^ homeostasis against metal-induced disturbances (Reed, 1990), it can affect the synthesis of thiol rich proteins such as MT (Kang *et al*., 1989). Therefore, decreases in the level of cellular GSH can be expected to give rise to a variety of functional changes, related either directly or indirectly to metal toxicity.

## Cadmium – uptake and toxicokinetics

Transition metals such as Cd are found in the marine environment as free ions, as well as in a variety of complexes with suspended particles and sediments. There are two major routes of metals exposure of water organisms mainly fish. Metal ions dissolved in the ambient water are absorbed through the gills (Alvarado *et al*., 2006), and other permeable body surfaces. Metals bound to solid particles are ingested, detached from their carrier particles in the digestive system and absorbed through the gut epithelium (Berntssen *et al*., 2001). The extent of these routes varies, depending partly on the chemical and physical characteristics of water and sediments (Wiclung and Runn, 1988). In the study of Wögrath and Psenner (1995), Arctic char (*Salvelinus alpinus*) from the small lakes in Tyrol (Austria) had high Cd concentrations in fish tissues, which contrasted with the low metal levels in the water. The real reason for this situation may be increased availability of Cd from the water due to its low alkalinity (Wögrath and Psenner, 1995). It is assumed that the low alkalinity may interfere with ionic transport across epithelia of freshwater fish resulting in the net accumulation of unwanted trace elements in their tissues (Haines, 1981). Uptake is also influenced by low pH and a lack of Ca^2+^ ions in the water (Spry and Wiener, 1991). As a first defence strategy the fish may try to avoid metal accumulation by secreting gill mucus that binds and immobilizes metals outside the organism (Handy and Eddy, 1990), subsequently changes that occur during chronic exposure range from the initial binding at the gill surface through to uptake into the blood, clearance from blood to tissues, mobilization of binding proteins, accumulation in tissues and finally excretion via the gills, liver and kidney (Hollis *et al*., 1999). Experimental data provide different information about Cd target. De Smet *et al*. (2001) presented that Cd concentrations increased in the different organs as a function of time and exposure concentration. The order of accumulation was kidney > liver > gills. Yudkovski *et al*. (2008) reported that Cd is accumulated in the liver and its further transportation to other organs is limited. It seems that the chronic exposure which may lead to steady state Cd hepatic levels requires long-term exposure to high Cd levels. Similarly Rose *et al*. (2006) specified that in fish most vulnerable organ to acute exposures is thought to be the gills. Liver and kidney are vulnerable organs during prolonged metal exposures, both from waterborne and dietary sources. On the other hand, McGeer *et al*. (2000) reported that Cd burden in rainbow trout (*Oncorhynchus mykiss)* chronically exposed to 3 µg Cd L^−1^ was primarily in the gill followed by stabilization of tissue metal concentration after 12 days, while the accumulation in liver was approximately linear after exposure to 3 g Cd L^−1^. Szebedinszky *et al*. (2001) observed an increased accumulation of Cd in the gills during a 30-day exposure to 2 µg Cd L^−1^, whereas an accumulation did not take place in the liver. Glynn and Olsson (1991) provided data that accumulation of new Cd internal tissues over the 72h of exposure illustrated that the kidney concentrations reach levels much higher than other tissues such as the liver or muscle. The situation was different in the kidney. This organ was the ultimate target for the accumulation of Cd in many fish species (Glynn *et al*., 1991). Lange *et al*. (2002) published that as the gills are the first site of uptake, new Cd accumulation into the gill was much higher than into the other organs. In their experiment new accumulation of Cd into the kidney was approximately 10-fold less than into the gill but the uptake trends were generally similar in that all fish-showed accumulation. New accumulation of Cd into the liver was approximately 50% of the uptake into the kidney (on a concentration basis). These discrepancies could be due to various factors influencing Cd uptake and distribution in organism. First of all, the characteristics of sentinel organism − species of fish, age, sex, living and feeding behaviour. It was evidenced that aged fish seem to accumulate more Cd in liver and kidney than juvenile fish (Olsvik *et al*., 2000). Smirnov *et al*. (2004) published that in some fish species, sex-related differences in tissue response to the input of metals such as Cd were observed. Santovito *et al*. (2000) reported that with respect to *Chionodraco hamatus*, *Trematomus bernacchi* liver accumulates more Cd. This finding correlated with the different feeding habits of the two species. In *T.bernacchi*, a benthic feeder, dietary uptake of Cd is likely to be the major route of assimilation, since this fish feeds on polychaetes, molluscs and epibenthic crustaceans, all species known to accumulate large amounts of metals. The mesopelagic *Ch. hamatus*, feeding on the small fish and krill has significantly lower contents of Cd. Analogous Köck *et al*. (1996) observed that the liver of *S. alpinus* is the main target organ for Cu and Zn, not for Cd.

## Cadmium – toxicodynamics

Variability in the target organ for Cd in fish is closely associated with endogenous fate of Cd in organism, that is affected by diversity in MT isoforms and GSH/GSSG level according to fish species. Cd exhibits a high affinity for thiol groups and disturbs many metabolic functions in cell (Belcastro *et al*., 2009). It reacts with free protein cysteine thiol groups that participate to various structural proteins, receptors and especially enzymes suppressing their catalytic activities (Friberg *et al*., 1986), and changing their structure. That disturbs various cellular metabolic processes (Jezierska and Witeska, 2001), and cause lipid peroxidation as a causative factor in metal-induced cell death (Stacey and Klaassen, 1981). It was proposed that the initial effect of non-specific Cd-binding to intracellular ligands (proteins) can be regarded as a toxic interaction and that subsequent detoxification of Cd by binding to protective molecule is a “rescue” phenomenon (Huang, 1993). Particularly, it is molecule of thiol, but it could be other substances. Interesting results were observed by Huang *et al*. (2007) that heavy metals including Cd, Hg and Pb are found to bind with the high molecular weight proteins (HMWP) in gills as well as in liver and kidney. Same results were reported by Kito *et al*. (1982a) that observed Cd binding with HMWP in gills and kidneys during the earlier periods of exposure and then with MT in the later stages (Huang *et al*., 2007). Similarly De Smet *et al*. (2001) reported that in gills, Cd existed in the high molecular weigh fraction (HMF) after 1 day of exposure and appeared after 4 days in the MT fraction (MTF). In liver, Cd was almost completely present in MT fraction, while in kidney appeared firstly in HMF and increased in MTF as time proceeded. The relative amounts of non-MT-bound Cd in the liver, gills and kidney were highest in the beginning of the Cd exposure and decreased with increasing intracellular Cd levels (Kito *et al*. 1982b). On the other hand, proteins with a lower affinity for the metal may also bind Cd, because of their relative high concentrations in the cell compared to MT. No significant binding seems to occur to low molecular weigh proteins with Mr <3000. Other cadmium-binding proteins will be the highest when low concentrations of Cd are present in the cell and MT concentrations are at their lowest levels (Kuroshima, 1992; De Smet *et al*., 2001). It may be mentioned the tripeptide GSH/GSSG by those low molecular weigh proteins, because increase of GSH/GSSG levels in all the tissue has been described as one of the protective mechanisms that fish adopt in the initial phases of exposure to aquatic pollutants (Stephensen *et al*., 2002). Cd in living body is bonded to thiol compound, at first moment to GSH/GSSG (Lange *et al*., 2002; Belcastro *et al*., 2009) and secondly to MT/MTLP (Santovito *et al*., 2000; Alvarado *et al*., 2005) and increasing of metal content in the body is accompanied enhanced level of thiols. GSH may work as a primary defence (Singhal *et al*., 1987). Same results were published by Klassen *et al*. (1999) that Cd is initially taken up by the liver, where it can bind with GSH and be excreted into bile. Alternatively, it can bind to MT and be stored (Norey *et al*., 1990). Some amount of Cd bound to MT leaks into plasma and subsequently is taken up by the kidney. Thomas *et al*. (1982) reported that chronic exposure to Cd caused a time and dose-dependent increase of hepatic GSH in fish like mullet (*Mugil cephalus*) or Atlantic croaker (*Micropogonias undulatus*) (Thomas *et al*., 1993), but elevated GSH level after the Cd treatment was followed by a subsequent decrease to initial level (Kuroshima, 1995). Olsson *et al*. (1998) mentioned that Cd treatment resulted in the induction of GSH as well MT-like protein in cells, but only in liver. Although, Chatterjee *et al*. (1984) observed that acute exposure to Cd was found to reduce GSH levels, and Tort *et al*. (1996) showed no significant effect of CdCl_2_ (1 mg/kg i.p.) on GSH in liver in *O. mykiss*. Nor increase of GSSG compared to GSH, in accordance to reduction of metal-induced oxidative stress, do not show explicit data. In study of Lange *et al*. (2002), no significant changes in GSH/GSSG ratio were detected, what may indicate that their experimental conditions of Cd exposure did not lead to oxidative stress in *O. mykiss*. Contrary, Winston *et al*. (1991) mentioned that Cd induces free radicals and lipid peroxidation, which may in turn depress renal functions. Ferreira *et al*. (2008) even referred that measuring of oxidative stress have been widely used in environmental monitoring regarding the presence of pollutants, including metals. The diversity is in selection of oxidative stress-induced molecules. Instead of GSH/GSSG they measured oxidative stress enzymes or lipid peroxidation rate. Similarly (Gravato *et al*., 2006) observed lipid peroxidation (LPO) of membrane lipides, or the oxidation of polyunsaturated fatty acids as the effect of exposure to metals. More consistent information concerning of MT are available. Since MT lower the cellular level of free metal ions, they are considered to provide protection against metal toxicity (Roesijadi, 1996). In normal conditions, cellular MT concentrations are low, but increase markedly on administration of certain metal ions (Belcastro *et al*., 2009). Recently data show that sublethal exposure to Cd resulted in MT induction in fish (Olsson *et al*., 1989; De Smet *et al*., 2001). However, if MT is present in a tissue, it binds metal at the beginning of exposure and no significant induction of synthesis is measured (Hamer, 1986). It was found that MT values appeared to be dose dependent and are maintained all throughout the experimental time from other study (Hamilton *et al*., 1987). Indeed, depuration produced a significant reduction in MT values (Alvarado *et al*., 2005). In the same study was shown that at the beginning of Cd exposure, most of the inherent MT pool was not completely saturated and uncompleted metal stimulated the production of MT mRNA and protein up to the end of the exposure time. There are several points that must be taken into account. Induction of MT synthesis is not linearly related to Cd dosage in either the liver or kidney (Bae *et al*., 1982). De Smet *et al*. (2001) reported that MT *de novo* synthesis in carp (*Cyprinus carpio*) according to Cd pollution is the most important in kidney. Induction of the MT synthesis occurs earlier in this organ compared to gills. Contrary, Santovito *et al*. (2000) published that the highest MT contents were found in liver in *T.bernacchi* and *Ch. hamatus.* Same results were observed in *S.alpinus* by Dallinger *et al*. (1996). Lange *et al*. (2002) presented that Cd exposure resulted in hepatic Cd accumulation and, as a consequence, in an induction of hepatic MT mRNA. The induction response was strictly correlated with hepatic metal dose. It seems that the sensitivity and organ specificity in MT gene induction by the specific heavy metal may be influenced by exposure condition including doses and manner of treatment. Although, Szebedinszky *et al*. (2001) presented that the response of MT mRNA varied with the metal or metal mixture, target organ and exposure time. Also the fish species plays role, how can be showed in results of De Boeck *et al*. (2003), in that induction of MT by metal was species- and tissue-specific in three freshwater fishes: rainbow trout (*Oncorhynchus mykiss*), common carp (*Cyprinus carpio*) and gibel carp (*Carassius auratus*). MT induction in gill has only been found in *C.auratus*. The choice of sentinel fish seems to be very important for the MT isoforms. Different fish species such as turbot (*Scotopthalmus maximus*), cod (*Gadus morhus*), pike (*Esox lucidus*) contain one form of MT, whereas *O.mykiss*, *C.auratus,* red sea bream (*Pagrus major*), channel catfish (*Ictalurus punctatus*) contain two forms of MT (Smirnov *et al*., 2004). Brouwer *et al*. (1992) concluded that Cd-MT-I and Cd-MT-III function primarily in the detoxification of metals, whereas Zn/Cu-MT-I, and Cu-MT-II perform important roles in metal homeostasis. From these observations result that measurement of total MT level is not suitable for conclusion of Cd impact. Exposure to metals seems to provoke a cascade of related cellular responses in connection to MT that involve the lysosomal system of hepatocytes (Olsson *et al*., 1998). Metals are neutralized in the form of MT-metal complexes that are transported to the lysosomes to minimize their possible toxic effects (Olsson *et al*., 1998). Lysosomes contain MT degradation products and serve as a final storage site of degraded MT and possibly, of other metal-binding proteins (Dallinger, 1995), hence sequestration of metal-MT complexes in lysosomes is considered as a general strategy (not exclusive for fishes) that contributes to reduce the potential toxicity of metals (Cajaraville *et al*., 1995). For that reason changes in lysosomal structure or in composition of their volume could be used as general marker of pollutant impact in field studies using fish as sentinel organisms (Lowe *et al*., 1981; Köhler *et al*., 1992; Cajaraville *et al*., 1995).

## Complications in use of thiols as biomarkers

Assessment of Cd contamination make more difficult also reality that metals are presented in combination in environment. Synergic or reverse effect to MT of each metal in ambient water, have still not be recognized. Yudkovski *et al*. (2008) also reported that MT transcript induction appeared only upon exposure to relatively high levels of Cd, not generally existing in the coastal environment or in rivers. However, acclimatization to elevated levels of trace elements may be achieved by altered uptake or elimination rates (Dallinger *et al*., 1996). At sites where metals are present and bio available at high concentrations, some species do not show increased MT concentrations, at least in some organs (Bervoets and Blust, 2003). It has been suggested that metal-acclimated fish exposed to a mixture of Cu, Zn and Cd, are less permeable to Cd and Zn, but more permeable to Cu, either as a result of changed metal-binding properties of the gill surfaces (McDonald and Wood, 1993). It was also presented that chronic sublethal exposure to Cu results in cross-acclimation to Cd in reduction of metal uptake (McGeer *et al*., 2007). There are other differences in MT content in relation with age and gender of fish. Concentrations of Cd, Zn-MT in the liver of *Rutilus rutilus* were found to increase with the animal's age (Bonwick *et al*., 1991). In the dab (*Limanda limanda)*, for example, the level of hepatic MT in females distinctly correlated with the accumulation of Zn, whereas that in males responded mainly to the mixture of Cu and Cd ions (Hylland *et al*., 1992). The sampling of fish often seems to be less controllable particularly as regards presence/absence, selection of size or weight categories, and the influence of these factors on the inducibility of fish MT is well-recognized (Tom and Auslander, 2005). Benthic organisms have been used in monitoring the toxicity of sediments, as they are directly affected by pollutants in the sediments (Zorita *et al*., 2007; Martín-Díaz *et al*., 2007), thus it could be used in assessment of metal contamination. Redeker *et al*. (2006) suggested that Cd-exposed oligochaetes, *Limnodrilus udekemianus* and *Tubifex tubifex*, could be considered as sensitive biomarkers of such exposure. Lecoeur *et al*. (2004) according to Pan and Zhang (2006) mentioned that MT concentration in hepatopancreas of *Charybdis japonica* showed a better dose-response and time-effect relationship with Cd concentration and duration of exposure. On the other hand, any MT-metal exposure relationship is easier to demonstrate in fish than in invertebrates, as there is less or no interference from bio mineralization processes, which are common in invertebrate trace metal detoxification (Mason and Jenkins, 1995). Considerably, in invertebrates, two major mechanisms of detoxification involving intracellular ligands have been well-documented: metal-binding to cytosolic compounds including MT (or MTLP) and bio mineralisation (Marigomez *et al*., 2002).

## Conclusion

Biomarkers, or biological responses of organisms to toxicant exposure, have been used for decades to indicate stress in aquatic organisms or the magnitude of environmental pollution (McCarthy and Shugart, 1990; Handy and Eddy, 1990). According to Depledge (1993) the definition of biomarker introduce that it is a biochemical, cellular, physiological or behavioural variation that can be measured in tissue or body fluid samples or at the level of whole organisms that provides evidence of exposure to and/or effects of, one or more chemical pollutants (and/or radiations). MT is now part of core suite of biomarkers recognized at European level and examined in the framework of biological effect quality assurance in monitoring programmes (BEQUALM) (Mathiessen, 2000). Nevertheless, literature data present many contraindications and inconsistencies in MT induction. The processes that generate these inconsistencies need to be understood as well as the relative influence of natural and contamination factors in order to validate the use of MT as biomarkers. Too many factors, influenced values of MT, limit the possibility of using MT concentration as a biomarker of metal exposure. Data on GSH and GSSG levels did not provide conclusive results. Probably, oxidative stress rising from metal exposure is reduced in reaction with antioxidant enzymes such as catalase (CAT) or superoxide dismutase (SOD), beside GSH/GSSG. Little information is available on the response of GSH and MT patterns to metal combinations, which are unique and not simply the cumulative effects of exposure to single metals. Likewise, the choice of the best species for monitoring is difficult. Recently, increased numbers of studies of thiol compounds have used crustaceans and other benthic invertebrates (Amiard *et al*. 2006; Won *et al*. 2008) for assessment of metal contamination, due to their permanent *in situ* benthic habitat and high absorption of metal. Nevertheless, some differences in induction of thiols between field and laboratory exposures for the invertebrates were also found (Correia *et al*., 2002). As the final place for MT storage, lysosomes may be taken into account. For future studies, it may help for eliciting details in thiol compounds function. It is necessary to perform other studies in field condition to obtain more information, to date, staying unexplained.
